# Trabeculated Myocardium in Hypertrophic Cardiomyopathy: Clinical Consequences

**DOI:** 10.3390/jcm9103171

**Published:** 2020-09-30

**Authors:** José David Casanova, Josefa González Carrillo, Jesús Martín Jiménez, Javier Cuenca Muñoz, Carmen Muñoz Esparza, Marcos Siguero Alvárez, Rubén Escribá, Esther Burillo Milla, José Luis de la Pompa, Ángel Raya, Juan Ramón Gimeno, María Sabater Molina, Gregorio Bernabé García

**Affiliations:** 1 Departamento de Ingeniería y Tecnología de Computadores, Universidad de Murcia, Espinardo, 30100 Murcia, Spain; josedavid.casanova@um.es (J.D.C.); jcuenca@um.es (J.C.M.); gbernabe@ditec.um.es (G.B.G.); 2 Unidad CSUR de Cardiopatías Familiares, Servicio de Cardiología, Hospital Universitario Virgen de la Arrixaca, Universidad de Murcia, El Palmar, 30120 Murcia, Spain; josegonca.alarcon@gmail.com (J.G.C.); jmj59c@gmail.com (J.M.J.); carmue83@gmail.com (C.M.E.); esbumi@hotmail.com (E.B.M.); mariasm@um.es (M.S.M.); 3 Instituto Murciano de Investigación Biosanitaria (IMIB), El Palmar, 30120 Murcia, Spain; 4 European Reference Networks (Guard-Heart), Red de Investigación Cardiovascular (CIBERCV), Instituto de Salud Carlos III, El Palmar, 30120 Murcia, Spain; 5 Intercellular Signalling in Cardiovascular Development and Disease Laboratory, Centro Nacional de Investigaciones Cardiovasculares (CNIC), Melchor Fernández Almagro 3, 28029 Madrid, Spain; marcos.siguero@cnic.es (M.S.A.); jlpompa@cnic.es (J.L.d.l.P.); 6 Centro de Investigación Biomédica en Red de Enfermedades Cardiovasculares (CIBERCV), Instituto de Salud Carlos III, 28029 Madrid, Spain; 7 Regenerative Medicine Program, Bellvitge Biomedical Research Institute (IDIBELL) and Program for Clinical Translation of Regenerative Medicine in Catalonia (P-CMRC), Hospital Duran i Reynals, Hospitalet de Llobregat, 08908 Barcelona, Spain; rescriba@idibell.cat (R.E.); araya@idibell.cat (Á.R.); 8 Centre for Networked Biomedical Research on Bioengineering, Biomaterials, and Nanomedicine (CIBER-BBN), 28029 Madrid, Spain; 9 Institució Catalana de Recerca i Estudis Avançats (ICREA), 08010 Barcelona, Spain

**Keywords:** advanced cardiac imaging, hypertrophic cardiomyopathy, myocardial disease, cardiac magnetic resonance, left ventricular non-compaction, trabeculas

## Abstract

Aims: Hypertrophic cardiomyopathy (HCM) is often accompanied by increased trabeculated myocardium (TM)—which clinical relevance is unknown. We aim to measure the left ventricular (LV) mass and proportion of trabeculation in an HCM population and to analyze its clinical implication. Methods and Results: We evaluated 211 patients with HCM (mean age 47.8 ± 16.3 years, 73.0% males) with cardiac magnetic resonance (CMR) studies. LV trabecular and compacted mass were measured using dedicated software for automatic delineation of borders. Mean compacted myocardium (CM) was 160.0 ± 62.0 g and trabecular myocardium (TM) 55.5 ± 18.7 g. The percentage of trabeculated myocardium (TM%) was 26.7% ± 6.4%. Females had significantly increased TM% compared to males (29.7 ± 7.2 vs. 25.6 ± 5.8, *p* < 0.0001). Patients with LVEF < 50% had significantly higher values of TM% (30.2% ± 6.0% vs. 26.6% ± 6.4%, *p* = 0.02). Multivariable analysis showed that female gender and neutral pattern of hypertrophy were directly associated with TM%, while dynamic obstruction, maximal wall thickness and LVEF% were inversely associated with TM%. There was no association between TM% with arterial hypertension, physical activity, or symptoms. Atrial fibrillation and severity of hypertrophy were the only variables associated with cardiovascular death. Multivariable analysis failed to demonstrate any correlation between TM% and arrhythmias. Conclusions: Approximately 25% of myocardium appears non-compacted and can automatically be measured in HCM series. Proportion of non-compacted myocardium is increased in female, non-obstructives, and in those with lower contractility. The amount of trabeculation might help to identify HCM patients prone to systolic heart failure.

## 1. Introduction

Hypertrophic cardiomyopathy (HCM) is a genetic cardiac disease characterized by clinical and prognostic heterogeneity [1,2,3,4]. The variable phenotypic expression and incomplete penetrance [5] have constituted an obstacle to obtain a full understanding of the consequences of the disease. HCM is mainly caused by mutations in genes encoding sarcomeric contractile proteins [6,7].

Left ventricular non-compaction (LVNC) is defined by an increase of trabeculations in the left ventricular endo-myocardium. Adult appearance of trabeculated myocardium may mainly be a consequence of an arrest in the embryologic process of compaction. 

Some authors have suggested that myocardial trabeculations can be acquired (although this hypothesis remains to be demonstrated) in response to an increase in cardiac load [8,9].

This entity was increasingly recognized with the development of high definition cardiac imaging. Although LVNC can be in isolation, an increase in hypertrabeculation often accompanies other genetic cardiomyopthies [10]. Despite early descriptions of LVNC in adults showed an increase in the rate of adverse outcomes, such as progression to heart failure, arrhythmias, and emboli [11,12], further analysis has showed inconsistent results [13,14,15]. 

The clinical relevance of the presence of hypertrabeculation in HCM is unknown. We aim to measure the left ventricular mass and proportion of trabeculation in a population of HCM patients. 

## 2. Materials and Methods

### 2.1. Study Sample

Patients recruited from an Inherited Cardiomyopathy Clinic—meeting international HCM criteria with an available good quality CMR study—were included. The first CMR of each patient was used for the study. Clinical and outcome data prospectively collected in a database was available for analysis. 

Patients were prospectively included, cardiac imaging and examinations were performed prospectively; symptoms, history of hypertension (HTN), regular physical exercise and arrhythmic events prior to the first evaluation were also recorded. HTN was defined following European Society of Hypertension and European Society of Cardiology recommendation [16]. 

Echocardiographic variables included: pattern of left ventricular hypertrophy (LVH) [17]; maximal left ventricular wall thickness (MWT); LV mass (Devereaux); LV systolic and diastolic diameters; left atrium diameter (LA); LV systolic and diastolic function (systolic dysfunction was considered if LVEF was <50%); left ventricular outflow tract gradient (LVOTG) and the presence of mitral regurgitation. LVOT obstruction was defined as LVOTG > 30 mmHg. 

**Figure 1:**
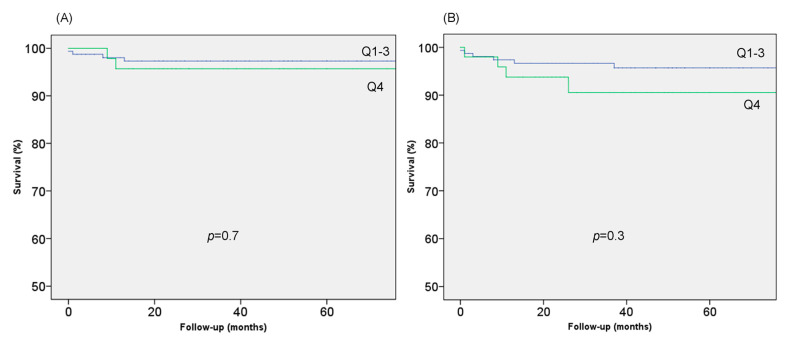
Survival for higher trabeculated myocardium (TM%) quartile (Kaplan Meier curves). (**A**) Sudden Death (SD) equivalent; (**B**) CV death.

All patients were offered a 24 h ECG-Holter and exercise test in order to complete sudden death (SD) risk stratification. Non-sustained ventricular tachycardia (NSVT) was assessed. Arrhythmic events and complications during follow up were also recorded. In the cohort, there were three resuscitated cardiac arrests (CA) and six appropriate implantable cardioverter defibrillator (ICD) discharges, which were computed as SD equivalent for survival estimates. Mean follow-up was 49.1 ± 37.1 months (Figure 1). 

All subjects gave their informed consent for inclusion before they participated in the study. The study was conducted in accordance with the Declaration of Helsinki, and the protocol was approved by the Ethics Committee of Clinical Research from Universitary Hospital Virgen de la Arrixaca (218/C/2015).

### 2.2. Trabecular Quantification

**Figure 2:**
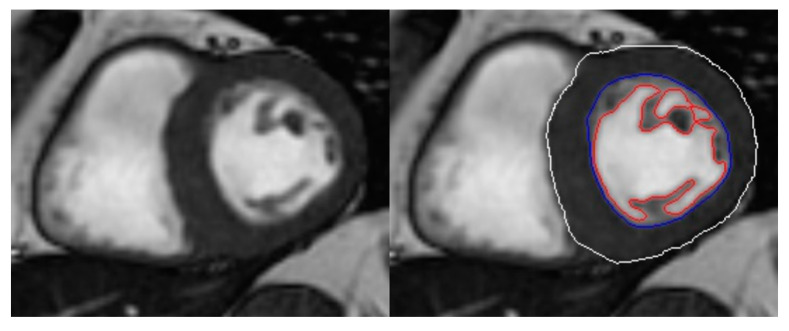
Example of automatic quantification of the compacted and trabeculated myocardium. **Left**: still diastolic coronal CMR image. **Right**: automatic border delineation of compacted and trabeculated myocardium from the same slice. White: epicardial border, blue: endocardial border, red: trabeculation border.

Magnetic resonance studies were performed in two hospitals with different scanners: SIGNA HDxt 1,5T: General Electric Systems, USA, post-processing software Advantage Workstation, AWA.3-08 and Achieva CV, Philips Medical Systems, Netherlands, (Philips Software workspace 2.6.3.2). A dedicated software tool for the automatic quantification and exact hyper-trabeculation degree of LVNC based on automatic delineation of the epicardial and endocardial borders of the LV and trabecular recesses was used [18] (see Supplementary Materials) (Figure 2).

Performance of the software was reviewed visually by one imaging cardiologist expert in order to rule out significant deviations. Additionally, delineation of the borders of 10 randomly selected cases was subjectively scored by two skilled cardiologists. The score went from 1 to 5, from large disagreement (1) to exact match (5). A total of 73 CMR slices were evaluated: 65 slices were scored with a five by the two and the remaining eight slices were classified with a four at least by one of the cardiology experts. The weighted kappa statistic showed an agreement between the two observers of 96.6% (kappa 0.76).

### 2.3. Statistical Analysis

Statistical analysis was performed using SPSS statistical software (version 21.0 IBM Corp. Chicago, IL, USA, 2012). Chi square, *t*-test, Anova and Pearson were used for comparison between groups where appropriate. Variables with *p* < 0.10 in univariate were included in the multivariate analysis. Additionally, those considered relevant to adjust the model were also included. Variables with redundant information or colinearity were excluded. Linear logistic regression was used for association between study variables (TM and TM%) and clinical variables. Kaplan Meier estimates and Cox regression analysis were used for survival analysis. *p*-values < 0.05 were considered statistically significant.

## 3. Results

The study population comprised 211 HCM patients (mean age 47.8 ± 16.3 years old, 154, 73% males).

Baseline clinical characteristics of patients included in the study regarding the quartile of trabeculation, are summarized in Table 1. Reason for diagnosis was symptoms in 88 (47.0%), screening in 51 (24.2%), incidental or unknown 71 (33.6%) and SD in 1 (0.5%). 43 (20.5%) were symptomatic, with 30 (14.2%) of patients in NYHA III-IV and 13 (6.3%) syncope. 42 (19.9%) had history of atrial fibrillation (AF), and 39 (19.8%) NSVT on ECG-Holter monitoring.

### 3.1. Echocardiography

Mean maximal LVH was 18.8 ± 5.1mm. 103 (50.2%) of patients with asymmetric LVH, and 64 (30.5%) demonstrated obstruction (Table 2).

### 3.2. LV Trabeculation and Demographics

Despite both trabeculated (TM) and compacted myocardial (CM) mass were increased in males compared to females, females had significantly higher TM% (29.7% vs. 25.6%, *p* < 0.0001). Indexed CM remained increased in males compared to females while TM mass was similar in males and females (Table 3).

There was no association between age and TM%. TM% was similar in 87 HTN versus 124 non-HTN patients with HCM (27.2 ± 6.1% vs. 26.4 ± 6.7%, *p* = 0.4) and in 23 patients who performed versus 181 who did not engage in intense physical activity (27.0 ± 4.6% vs. 26.7 ± 6.6%, *p* = 0.7). 

### 3.3. LV Trabeculation and Morphological Findings

**Figure 3:**
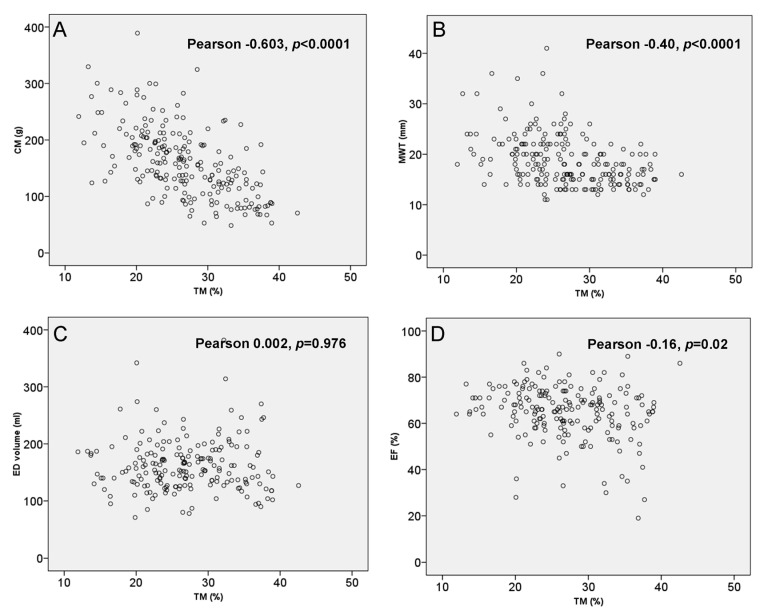
Correlation between TM%, (**A**) CM, (**B**) MWT (from echocardiography), (**C**) ED volume, and (**D**) EF%.

Correlation between TM% and main variables from CMR are illustrated in Figure 3. An inverse correlation between MWT and TM% was observed (Pearson −0.40, *p* < 0.0001) (Figure 3B). Patients with severe LVH (MWT ≥ 30mm) had similar TM but significantly lower TM% compared to non-severe ones (20.0 ± 4.9% vs. 27.0 ± 6.3%, *p* = 0.002).

Patients with obstruction had increased CM mass (and indexed CM mass) but reduced TM% compared to non-obstructives (184.3 ± 68.0 g vs. 149.6 ± 56.4 g, *p* < 0.0001 and 24.9 ± 27.6% vs. 27.6 ± 6.3%, *p* < 0.0001, for CM and TM%, respectively). Neutral pattern had an increased TM% (28.4 ± 5.9% vs. 25.8 ± 6.7%, *p* = 0.004), while septal reverse and apical pattern of hypertrophy had significantly less TM% (22.4 ± 6.2% vs. 27.6 ± 6.2%, *p* < 0.0001, and 22.8 ± 3.9% vs. 27.1 ± 6.5%, *p* = 0.01). There was no correlation between left atrium diameter and TM%. 

No correlation was found between end-diastolic and end-systolic LV volumes from CMR and TM% (Figure 3C). An inverse correlation was demonstrated between LVEF% and TM% (Pearson −0.16, *p* = 0.02) (Figure 3D). Patients with LVEF < 50% had higher TM% values (30.2 ± 6.0% vs. 26.6 ± 6.4%, *p* = 0.02). 

Late gadolinium enhancement (LGE) was associated with CM, TM and TM% (including indexed parameters). Of the patients, 106 (54.1%) with positive LGE had significantly higher CM mass (179.4 ± 61.8 g vs. 138.4 ± 56.9 g, *p* < 0.0001), higher TM (58.3 ± 18.5 vs. 52.3 ± 19.1 g, *p* = 0.03) but lower TM% (25.3 ± 5.9% vs. 28.3 ± 6.8%, 0.001), compared to the 90 (45.9%) with no LGE.

### 3.4. LV Trabeculation and Clinical Findings 

TM% was not related with symptoms. Patients with dyspnea (NYHA III-IV) or syncope had similar TM and TM%. There was no difference in TM% in patients with or without AF. There were only 6 (2.8%) patients with sustained ventricular tachycardia who had a significantly lower TM% (19.8 ± 6.0 vs. 26.9 ± 6.3, *p* = 0.007).

Nine (4.3%) patients who required pacemaker implantation had significantly lower TM% (20.2 ± 5.0% vs. 27.0 ± 6.3%, *p* = 0.002). 26 (12.3%) patients who subsequently underwent ICD implantation seemed to have also lower TM% (24.5 ± 6.3% vs. 27.0 ± 6.4%, *p* = 0.058) (Table 4).

Eleven (5.2%) patients who developed a stroke had lower TM% than those who did not (22.7 ± 7.9% vs. 26.9 ± 6.3%, *p* = 0.03). 

In total, 12 (5.7%) patients had at least one major event over a mean 49.1 ± 37.1 months of follow-up: three patients died suddenly, one had a resuscitated cardiac arrest and four had appropriate ICD therapies. There was one heart failure death, one stroke related death, one procedure related death, and one heart transplant. 

The eight patients with SD (or equivalent) had significantly higher CM (224.8 ± 77.6 g vs. 157.5 ± 60.1 g, *p* = 0.02) and TM mass (75.1 ± 23.1 g vs. 54.7 ± 18.2 g, *p* = 0.002) but similar TM% (25.4 ± 5.1% vs. 26.8 ± 6.5%, *p* = 0.5). TM% was not associated with the combined major outcome. 

### 3.5. Multivariable Analysis

Female gender, MWT, neutral pattern of hypertrophy, obstruction, and LVEF were associated with TM% on multivariable analysis which included morphologic and demographic predictors. Age, HTN, physical exercise, left atrium diameter, or the presence of LGE were not associated with TM% (Table 5).

Symptoms (syncope or dyspnea) were not associated with TM% in a model with demographic, main morphological variables and symptoms. AF and NSVT on Holter was neither associated with TM%.

On multivariable analysis TM% was not associated with cardiovascular (CV) death or equivalent. MWT and AF were the only two variables associated with CV death. 

In the presence of CM mass calculated from CMR, MWT lost his value in the prediction of CV death. In this model, AF remained significantly associated (and a trend of TM%) (Table 6).

Severity of wall thickness, measured as MWT or CM mass were the strongest predictors of CV death and SD. 

## 4. Discussion

This is the first study to systematically quantify LV trabeculation in an HCM population. The main finding from our study was that a clinical profile of patients with HCM with increased trabeculations may exist. Some of the features were predominant female gender, and non-obstructive forms with systolic impairment. There was no association between TM with age, ventricular volumes, or LGE. Higher degree of trabeculation in HCM was associated with LVEF below 50% which might precede development of systolic heart failure [19]. Degree of trabeculations do not seem to associate to an increase arrhythmic risk in HCM. A longer follow up is needed confirm these findings.

Jacquier et al. [20] manually measured the TM mass in a group of patients with isolated non-compaction, dilated cardiomyopathy, HCM, and controls. A threshold of 20% of TM was proposed as a good cutoff for the diagnosis of LVNC (S and E >93%). Sixteen LVNC patients had an average of trabeculated mass of 32%, compared to 11% to 12% of DCM, HCM and controls. In this paper, the 16 patients with HCM had a mean CM of 216 g, which is significantly higher to the one measured in our study (160.0 ± 62.0 g). On the contrary, trabeculated myocardial mass was lower in that paper compared to our estimation from 211 patients (28 g vs. 55.5 ± 18.7 g). In Jacquier’s paper, papillary muscles were manually excluded from the TM, while in our automatic measurement this was included. Observed differences cannot solely be explained by different methodology [21].

The percentage of trabeculated myocardium showed in this large series of HCM (26.7 ± 6.4%) patients demonstrate a clear increase from that observed in controls, which suggests that LVNC or hypertrabeculation is part of the clinical phenotype of HCM. In an earlier paper from our group using the same software, a cutoff of 27% MT showed to have a good accuracy for differentiation of LVNC [22].

In contrast to the fractal analysis recently published by Captur et al. [23], based on tortuosity measured by pixelation of the line of the endocardial border, our software provides easy to understand clinical measurements of CM and TM mass. This computationally-assisted method could save valuable diagnostic time compared with traditional processing, thus minimizing the possibility of human error. We demonstrate also in this population of HCM patients, a good performance of the software for quantification of hypertrabeculation based on the automatic delineation of borders from CMR diastolic images [24]. 

Trabeculation in its different forms, including deep crypts and clefts has been suggested to be a pre-diagnostic (maybe congenital) feature in carriers of HCM associated mutations [25]. While other authors suggest an acquired mechanism [26], from observations of increases of trabeculation in high demanding conditions such as in athletes and pregnancy, the mechanism of how this occurs is however unknown. Moreover, a reversion of the phenotype with the normalization in cardiac load after delivery, has been shown in one of those studies [27]. We have failed to show any association between the degree of physical activity, presence of HTN, or obstruction in the magnitude of trabeculations in our series of HCM patients. 

In contrast with an early paper of a dilated cardiomyopathy series [28], which suggested a higher rate of stroke in patients with LVNC, we also failed to demonstrate association between embolic events in higher trabeculated ventricles with HCM.

To finalize, we want to highlight the need of a quantification of trabeculation in order to define the limits between normality and abnormality and to establish the role of non-compaction in various cardiac conditions. Our software has demonstrated to be useful not only for automatic quantification of TM but also for CM. In this regard, in our study, CM mass measured by the software was the strongest predictor of the outcome above the traditional maximal wall thickness taken from echocardiography. The latter supports the use of global assessment of left ventricular CM and TM mass to replace focal measurements of wall thickness in risk algorithms.

## 5. Limitations

Differences in the characteristics of our series regarding distribution of the pattern of hypertrophy, value of LVH mass, proportion of obstruction or percentage of LGE might be due to the age of the cohort, which is relatively young (mean 44 years old), the inclusion of a ~34% of relatives (as compared to probands) or the relatively high percentage of patients diagnosed through family screening (25%) or incidentally (28%). Further studies are warranted to verify our findings.

Despite the number of individuals being relatively large, only a few patients developed CV events. Survival analysis is then limited, and results should be taken carefully. Patients with devices were ruled out from CMR and some high-risk patients were excluded. Repeated CMR studies were not available and changes over follow-up in CM and TM could not be explored. 

Genetic information was available in 88/140 (62.9%) of the probands included in the cohort. Genetic testing yield was 43/88 (48.9%). Further analysis of the association between genetic results and morphologic and clinical findings was not performed due to the incompleteness of the genetic information.

## 6. Conclusions

A significant proportion (~25%) of myocardium appears non-compacted and can be automatically measured in HCM series. There is a clinical profile of patients with HCM with increased trabeculations. Proportion of non-compacted myocardium is increased in female, non-obstructives, and in those with lower contractility. The amount of trabeculation might help to identify HCM patients prone to systolic heart failure. Amount of trabeculation does not seem to associate with arrhythmias.

## Figures and Tables

**Table 1:** Demographics and clinical characteristics per trabeculation quartile *.

		Q1	Q2	Q3	Q4	Total	Sig. (*p*)
n		54 (25.6)	51 (24.2)	54 (25.6)	52 (24.6)	211 (100.0)	
Age at diagnosis		45.8 ± 15.5	42.5 ± 17.8	43.2 ± 17.0	45.9 ± 18.8	44.4 ± 17.2	0.668
Age at CMR		50.6 ± 14.9	48.2 ± 17.9	47.4 ± 16.5	53.1 ± 15.2	49.8 ± 16.2	0.267
Proband		41 (75.9)	32 (62.7)	40 (74.1)	28 (53.8)	141 (66.8)	0.059
Sex	Male	38 (70.4)	39 (76.5)	39 (72.2)	38 (73.1)	154 (73)	0.880
	Female	16 (29.6)	12 (23.5)	15 (27.8)	14 (26.9)	57 (27)	0.880
Reason for diagnosis	Incidental	18 (33.3)	18 (29.4)	16 (29.6)	12 (23.1)	61 (28.9)	0.275
	Symptoms	25 (46.3)	19 (37.3)	24 (44.4)	20 (38.5)	88 (41.7)	0.586
	Screening	9 (16.7)	15 (29.4)	13 (24.1)	14 (26.9)	51 (24.2)	0.326
	SD	1 (1.9)	0 (0.0)	0 (0.0)	0 (0.0)	1 (0.5)	0.183
Genetics	Positive	20 (37.0)	18 (35.3)	26 (48.1)	21 (40.4)	85 (40.3)	0.537
	MYBPC3	10 (50.0)	11 (61.1)	20 (76.9)	14 (66.7)	55 (64.7)	0.136
	MYH7	7 (35.0)	3 (16.7)	1 (3.8)	3 (14.3)	14 (14.5)	0.135
	Other	3 (15.0)	4 (22.2)	5 (19.2)	3 (14.3)	13 (15.3)	0.866
HTN		20 (37.0)	23 (45.1)	22 (40.7)	22 (42.3)	87 (41.2)	0.698
Physical activity		13 (24.5)	16 (32.0)	27 (50.9)	23 (45.1)	79 (38.2)	0.007
Athlete		2 (3.8)	5 (10.0)	11 (20.8)	5 (9.8)	23 (11.1)	0.131
NYHA	I	32 (61.5)	36 (73.5)	37 (69.8)	39 (76.5)	144 (70.2)	0.147
	II	16 (30.8)	13 (26.5)	13 (24.5)	9 (17.6)	51 (24.9)	0.126
	III	3 (5.8)	0 (0.0)	2 (3.8)	2 (3.9)	7 (3.4)	0.859
	IV	1 (1.9)	0 (0.0)	0 (0.0)	1 (2.0)	2 (1.0)	0.998
Syncope		4 (7.7)	4 (8.2)	3 (5.7)	2 (3.8)	13 (6.3)	0.356
Atrial Fibrillation		11 (20.4)	10 (19.6)	12 (22.2)	9 (17.3)	42 (19.9)	0.794
SD RISK FACTOR							
FHSCD		4 (7.4)	10 (19.6)	5 (9.3)	7 (13.5)	26 (12.3)	0.684
ABPR		6 (14.3)	5 (12.8)	9 (20.9)	3 (7.1)	23 (13.9)	0.580
NSVT		16 (30.2)	9 (18.4)	9 (18.8)	5 (10.6)	39 (19.8)	0.020
Sustained VT		2 (3.7)	3 (5.9)	1 (1.9)	0 (0.0)	6 (2.8)	0.144

* Distribution of quartiles is adjusted by gender. FHSCD: Family history of sudden cardiac death (aged < 45 years old); ABPR: Abnormal blood pressure response on exercise test; HTN: Hypertension; NSVT: Non-sustained ventricular tachycardia; SD, sudden death; physical activity: Significant regular physical activity of >70% maximal exercise, for at least 3 h per week during the last 2 years before first evaluation. Athlete: Intense regular physical activity of >70% of maximal exercise, for at least 5 h per week during the last 2 years before first evaluation.

**Table 2:** Morphological and functional characteristics per trabeculation quartiles *.

		Q1	Q2	Q3	Q4	Total	Sig. (*p*)
Max LVH (mm)		21.1 (5.1)	19.5 (6.3)	18.1 (4.0)	16.3 (3.1)	18.8 (5.1)	<0.001
Pattern of hypertrophy	ASH	27 (51.9)	21 (44.7)	25 (46.3)	30 (57.7)	103 (50.2)	0.551
	Concentric	10 (19.2)	11 (23.4)	17 (31.5)	13 (25.0)	51 (24.9)	0.537
	Apical	5 (9.6)	7 (14.9)	3 (5.6)	0 (0.0)	15 (7.3)	0.032
	Reverse	14 (28.0)	7 (14.9)	6 (11.1)	3 (5.8)	30 (14.8)	0.012
	Neutro	13 (26.0)	18 (38.3)	25 (46.3)	29 (55.8)	85 (41.9)	0.018
	Sigmoid	3 (6.0)	3 (6.4)	4 (7.4)	4 (7.7)	14 (6.9)	0.985
Obstruction		25 (47.2)	13 (25.5)	16 (29.6)	10 (19.2)	64 (30.5)	0.005
Severe Obstruction		12 (22.2)	6 (12.0)	5 (9.3)	4 (8.0)	27 (13.0)	0.027
LVED (ml)		158.1 (49.0)	154.5 (40.1)	165.1 (32.9)	171.5 (57.4)	162.5 (46.0)	0.281
LVES (ml)		53.7 (37.6)	51.4 (20.7)	58.5 (25.3)	71.9 (47.3)	59.1 (35.2)	0.019
LVEF (%)		67.9 (10.7)	67.2 (7.5)	64.5 (11.4)	60.1 (14.4)	64.8 (11.7)	0.004
LVEF < 50%		2 (4.2)	3 (6.3)	6 (11.8)	8 (16.3)	19 (9.7%)	0.023
LGE		31 (63.3)	31 (63.3)	27 (55.1)	17 (34.7)	106 (54.1)	0.003

* Distribution of quartiles is adjusted by gender. Max LVH: maximal left ventricular hypertrophy (mm) from echocardiography, Obstruction: Left ventricular out flow tract gradient at rest or Valsalva of >30 mmHg estimated from echocardiography, Severe obstruction: Left ventricular out flow tract gradient at rest or Valsalva of >30 mmHg estimated from echocardiography. LVED: left ventricular end diastolic volume (ml) from CMR, LVES: Left ventricular end systolic volume (ml) from CMR, LVEF: Left ventricular ejection fraction (%) from CMR, LGE: Late gadolinium enhancement on CMR.

**Table 3:** Cardiac magnetic resonance trabecular parameters by gender.

	Female	Male	Total	Sig. (*p*)
CM	124.8 ± 55.5	173.1 ± 59.3	160.0 ± 62.0	<0.0001
TM	49.8 ± 18.5	57.6 ± 18.5	55.5 ± 18.7	0.007
TM%	29.7 ± 7.2	25.6 ± 5.8	26.7 ± 6.4	<0.0001
CM (indexed)	72.2 ± 32.0	87.9 ± 30.1	83.6 ± 31.3	0.001
TM (indexed)	29.1 ± 11.6	29.1 ± 9.3	29.1 ± 10.0	0.977

CM: Compacted myocardium (g). TM: Trabeculated myocardium (g). TM%: Proportion of trabeculated myocardium.

**Table 4:** Devices and disease related outcomes per trabeculation quartiles *.

	Q1	Q2	Q3	Q4	Total	Sig. (*p*)
Pacemaker	7 (13.0)	1 (2.0)	0 (0.0)	1 (1.9)	9 (4.3)	0.004
ICD	9 (16.7)	6 (11.8)	8 (14.8)	3 (5.8)	26 (12.3)	0.145
Sustained VT	2 (3.7)	3 (5.9)	1 (1.9)	0 (0.0)	6 (2.8)	0.144
Stroke	7 (13.0)	1 (2.0)	2 (3.7)	1 (1.9)	11 (5.3)	0.020
Transplant	0 (0.0)	0 (0.0)	0 (0.0)	1 (1.9)	1 (0.5)	0.178
SD	2 (3.7)	3 (5.9)	1 (1.9)	2 (3.8)	8 (3.8)	0.762
HF Death	1 (1.9)	0 (0.0)	0 (0.0)	1 (1.9)	2 (0.9)	0.993
CV death	3 (5.6)	3 (5.9)	2 (3.7)	4 (7.7)	12 (5.7)	0.774

* Distribution of quartiles is adjusted by gender. ICD, implantable cardioverter defibrillator, VT: Ventricular tachycardia, SD: Sudden death (includes sudden death, resuscitated cardiac arrest and ICD appropriate therapy), HF death: Heart failure death (included heart failure death and cardiac transplant), CV death: Cardiovascular death (includes SD, HF death, stroke related death and other disease related deaths).

**Table 5:** Variables associated with trabeculated myocardium (%) on multivariable analysis.

	Beta	95% CI	Sig. (*p*)
Female	4.294	(2.466, 6.122)	<0.0001
LVEF (%)	−0.124	(−0.195, −0.053)	0.0007
Obstruction (absence)	1.819	(0.043, 3.595)	0.0448
MWT (mm)	−0.423	(−0.605, −0.242)	<0.0001
Pattern (neutre)	1.725	(0.021, 3.429)	0.0473

**Table 6:** Variables associated with cardiovascular death (or equivalent) on multivariable analysis.

	Exp (B)	95% CI	Sig. (*p*)
Compacted myocardium (g)	1.011	(1.002, 1.021)	0.0197
Atrial Fibrillation	8.914	(2.267, 35.050)	0.0017

## Supplementary Materials

The following are available online at https://www.mdpi.com/2077-0383/9/10/3171/s1, S1 File: Magnetic resonance studies.
