# Supplementary material for: Trabeculated Myocardium in Hypertrophic Cardiomyopathy: Clinical Consequences
Source: J Clin Med. 2020 Sep 30;9(10):3171. doi: 10.3390/jcm9103171 (PMC7600439; doi:10.3390/jcm9103171)
Supplement: Supplementary file 1 [file jcm-09-03171-s001.pdf]

SUPPLEMENTARY MATERIAL

Magnetic resonance studies were performed in two hospitals with different scanners:

- Magnetic Resonance of the Southeast. Affidea (Spain). The analysis was performed using a personal computer and semi-automated software (Advantage Workstation, AW4.3-08). The magnetic resonance studies were performed with a 1.5-T scanner (SIGNA HDxt 1,5: General Electric Systems, USA). The images were obtained in synchronization with the ECG and in apnea. The left ventricle function was evaluated with a free precision in standard stable balance (repetition interval of 3.8 ms, echo time of 1.7 ms, flip of 60, matrix of 224 × 224, echo train length of 23, cutting thickness of 8 mm, space between slices of 2 mm, with 20 phases). The post-processing to determine the ventricular parameters was performed with a workstation provided by the manufacturer (Advantage Workstation, AW4.3-08) in the Hospital Mesa de Castillo in Murcia (Spain).
- University Hospital Virgen de la Arrixaca in Murcia (Spain). The analysis was performed using a personal computer and semi-automated software (Philips, Software work space 2.6.3.2). The magnetic resonance studies were performed with a 1.5-T magnet (Achieva CV, Philips Medical Systems, Netherlands). The images were obtained in synchronization with the ECG and in apnea. The left ventricle function was evaluated with a free precision in standard stable balance (repetition interval of 3.3 ms, echo time of 1.7 ms, flip of 60, matrix of 192 × 256, echo train length of 23, cutting thickness of 8 mm, space between slices of 2 mm, with 20 phases). The post-processing to determine the ventricular parameters was performed with an independent workstation provided by the manufacturer (View-Forum 6.3, Philip Medical System).

CMR cine images were reviewed by two experienced investigators independently. Short axis slices, from the apex to the mitral annulus in end-diastole were analyzed. A standard protocol was used for measurements of LV volumes and wall thickness. A dedicated software tool for the automatic quantification and exact hyper-trabeculation degree of left ventricular non-compaction (LVNC) based on automatic delineation of the epicardial and endocardial borders of the left ventricular (LV) and trabecular recesses was used: delineation of the endocardial border, endocardial compacted layer, and pericardial border was performed automatically. The trabecular zones are detected inside and around the LV cavity. The software produces measurements of area, volume, and estimates of mass of compacted and noncompacted LV myocardium per slice, and total LV. All measurements are presented as absolute values and are indexed by body surface area. The proportion of trabeculated mass from total LV mass was also calculated (1).

1. Bernabé, G., Casanova, J.D., Cuenca, J., González-Carrillo, J. A self-optimized software tool for quantifying the degree of left ventricle hyper-trabeculation. J Supercomput (2019) 75: 1625.
